# The Advances and Utility of Artificial Intelligence and Robotics in Regional Anesthesia: An Overview of Recent Developments

**DOI:** 10.7759/cureus.44306

**Published:** 2023-08-29

**Authors:** Arunabha Karmakar, Muhammad Jaffar Khan, Mohamed El-Fatih Abdul-Rahman, Umair Shahid

**Affiliations:** 1 Anesthesiology and Critical Care, Hamad Medical Corporation, Doha, QAT

**Keywords:** artificial intelligence and robotics in healthcare, pain management, ultrasound-guided nerve block, regional anesthesia, robotics, artificial intelligence

## Abstract

The integration of artificial intelligence (AI) and robotics in regional anesthesia has brought about transformative changes in acute pain management for surgical procedures. This review explores the evolving landscape of AI and robotics applications in regional anesthesia, outlining their potential benefits, challenges, and ethical considerations. AI-driven pain assessment, real-time guidance for needle placement during nerve blocks, and predictive modeling solutions for nerve blocks have the potential to enhance procedural precision and improve patient outcomes. Robotic technology aids in accurate needle insertion, reducing complications and improving pain relief. This review also highlights the ethical and safety considerations surrounding AI implementation, emphasizing data security and professional training. While challenges such as costs and regulatory hurdles exist, ongoing research and clinical trials demonstrate the practical utility of these technologies. In conclusion, AI and robotics have the potential to reshape regional anesthesia practice, ultimately improving patient care and procedural accuracy in pain management.

## Introduction and background

Artificial intelligence (AI) at its root describes the ability of a machine to learn and act as a human would. Artificial intelligence refers to the study of algorithms that enable machines to reason, perform tasks such as problem-solving, recognize objects/words, infer world states, and make decisions. The previous iterations of AI were limited, being able to only replicate human behavior rather than being autonomous, self-learning, and adapting. Due to exponential improvement in computer science technology especially in computational speed due to faster chips, AI systems have now passed a pivotal stage in their evolution. Modern AI has a computing and analytical ability that outstrips most human minds, with the added advantage of being non-fatigable. These features make it a strong contender in the current human workforce for performing both routine and complex tasks. Unlike their versions from the past that only imitated human behavior, current AI can be used in multiple healthcare domains with performance benefits to the overall health system. Its impact on regional anesthesia and pain management is the focus of this review.

**Figure 1:**
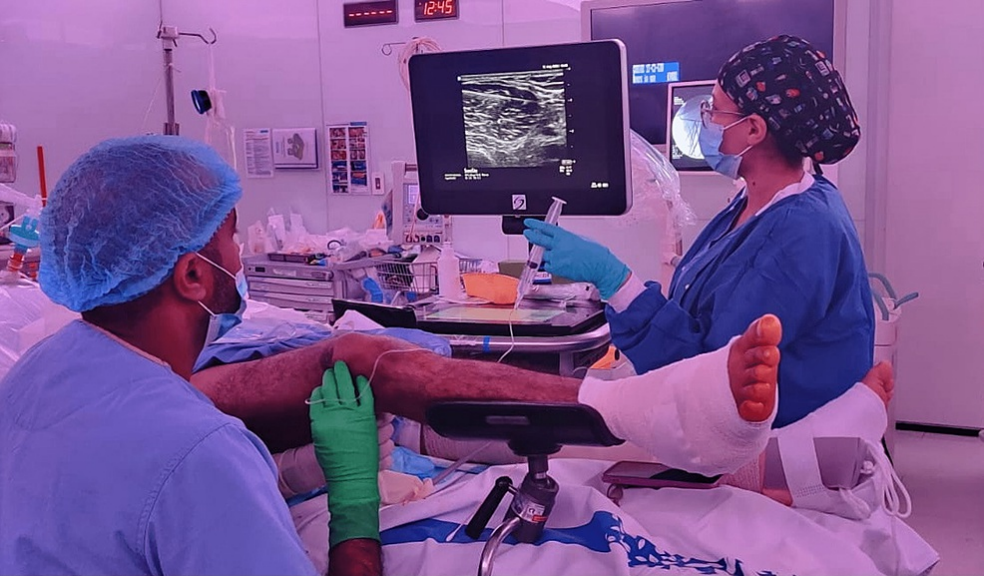
Performing an ultrasound-guided nerve block is a multitasking process. The image is the original work of the author.

Regional anesthesia is a specialized field of anesthesia involving the visualization of specific anatomical structures and selective nerve blockade around these structures. Such a procedure requires practiced skills developed with training and experience [1]. The safe independent performance of a nerve block currently takes years for a trainee anesthetist to master (Figure 1). Thus, the safe and efficient performance of nerve blocks and training to perform regional anesthesia hold promise for improvement by technology. A study looking at 520 peripheral nerve blocks (PNB) performed by six anesthesia residents identified five quality-compromising behaviors [2]: failure to recognize the maldistribution of local anesthesia, failure to recognize the intramuscular location of the needle tip, fatigue, failure to synchronize the laterality of the patient with the laterality of the image, and the poor choice of needle insertion site and angle concerning the probe. They identified failure to visualize the needle before advancement and unintentional probe movement as the two most common errors.

AI can rapidly peruse big data including patient, performer, procedure, and drug-related details; formulate strategic steps to correctly perform a given nerve block; act as a supervisor or assistant to a trainee; and rapidly identify scenarios to improve block success.

**Figure 2:**
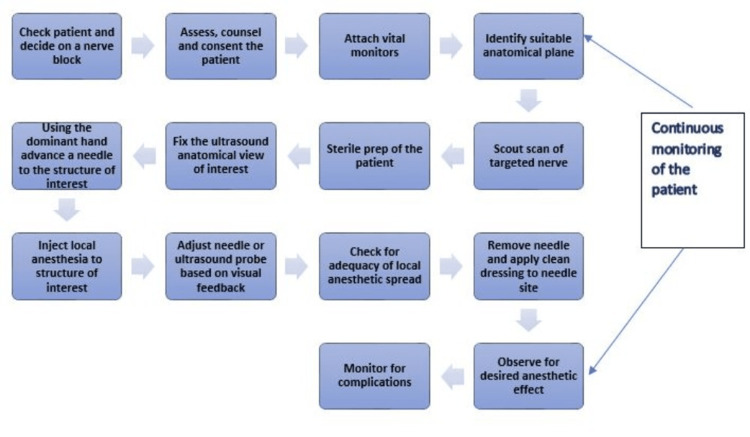
A safe performance of a peripheral nerve block is characterized by multiple sequential steps, all of which must be performed adequately. The use of artificial intelligence (AI) systems allows increased precision and efficiency in this interaction.

The steps of performing a nerve block are shown below (Figure 2). Machines can assist in each of these steps.

Machine assistance also allows increased human efficiency and the reduction of human error. This improves the success rate of a PNB and reduces the complication rate. In a few cases, it may also be for the ergonomic benefit of the healthcare provider. Hence, this review studies the recent advancement in AI technology in the field of regional anesthesia and its potential benefits including the identification of anatomical structures, education and training, and patient safety and outcome.

## Review

Method

We conducted a literature search via PubMed, Scopus, and Google Scholar, using the combinations of the following keywords: artificial intelligence, robotics, technology, regional anesthesia, ultrasound (US)-guided nerve block, and pain management without date restrictions. The search resulted in a variety of articles, including reviews, clinical trials, editorials, and observational research. After extracting articles from databases, we also conducted a manual search to screen more relevant literature. Article inclusion and exclusion were determined by their relevance to the application of AI or robotics in regional anesthesia and pain management.

This review delves into the integration of artificial intelligence and robotics in regional anesthesia, highlighting their importance and the call for advancements. It is crucial to understand that while the paper aims to cover recent AI and robotics progress in this field, it cannot encompass all aspects of evolving trends. The paper's limitations arise from its focus on specific advancements, missing out on other ongoing developments. Furthermore, due to constraints, it was not possible to comprehensively explore practicalities, global availability, usage, and the associated pros and cons.

AI-assisted technology in regional anesthesia

Real-Time Anatomical Guidance

The visualization of specific anatomical structures in modern regional anesthesia practice is done by bedside ultrasonography or fluoroscopy. The practitioner interprets the images and manually controls the probe or patient position and instrument to acquire real-time anatomical details. The spatial interpretation of these images in real time needs strong visual coordination and the ability to have a three-dimensional (3D) mental map of the human body. Following satisfactory image acquisition, the practitioner intervenes at the foci of interest by the insertion of a needle or catheter and the safe administration of a pharmacological agent. The process is characterized by real-time feedback from the monitor and imaging equipment to adjust for errors or mistakes made.

Human visual acuity and the speed of interpretation of images are limited by experience [3]. Additionally, most ultrasound images depict tissue structures on the scales of white and black. The human interpreter depends on nonintuitive image processing, which takes years to master. For example, the visualization of pulsation is used to distinguish an artery from a vein, both of which can appear as round hypoechoic structures. Another example is tracing the path of a nerve as it follows a known trajectory or accompanies a blood vessel, such as identifying the median nerve, which lies medial to the brachial artery and vein when examining the cubital fossa. Such interpretation can be prone to error. Errors can arise from the improper interpretation of the anatomical structure, the presence of acoustic artifacts, difficulty in needle visualization, and patient factors such as muscle atrophy, obesity, and tissue edema [4]. With sufficient practice (most regional anesthesia fellowships last one year followed by a period of supervised practice), a trained anesthetist can safely perform common nerve blocks. Even then, the errors of human judgment and human fatiguability are factors that are challenging to completely overcome.

Fast intelligent processing of anatomy helps improve efficiency, accuracy, and ergonomics (Figure 2). The eye of an anesthetist is better cued in when image processing by AI assists by highlighting important structural elements by labeling or color overlays [5,6], which are easily decipherable by a human mind. Needle tracking, checking the spread of local anesthetic, and monitoring patient comfort can all be assisted by an AI as well.

**Figure 3:**
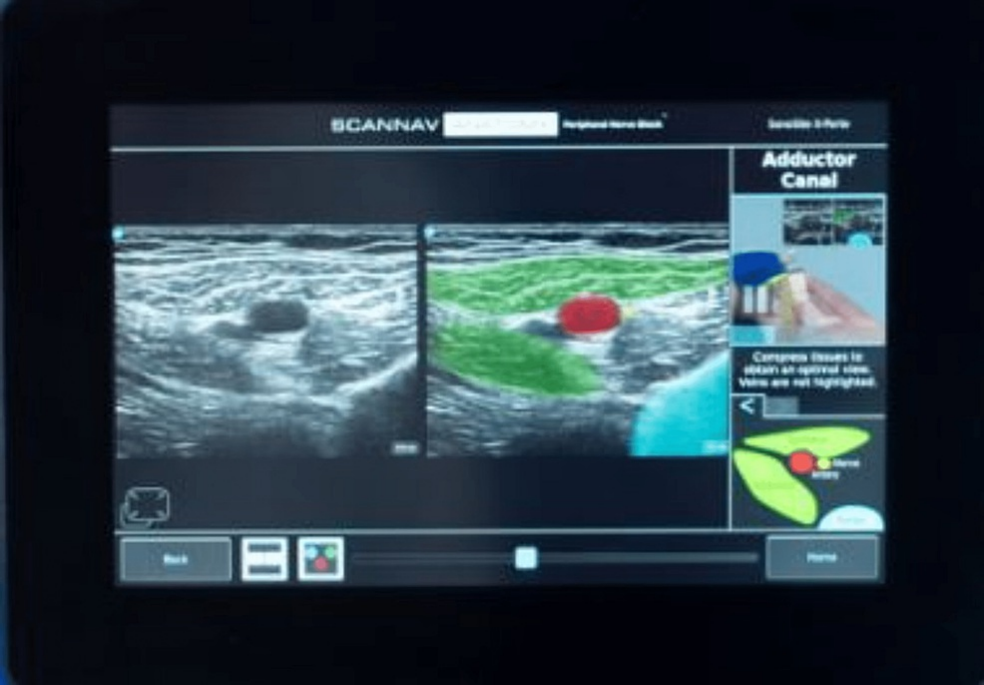
Ultrasound scanner with ScanNav™ Anatomy PNB software. Intelligent Ultrasound Simulation is the trading name of MedaPhor Ltd. (ISO 13485 Certified, Copyright© 2023 Intelligent Ultrasound).

**Figure 4:**
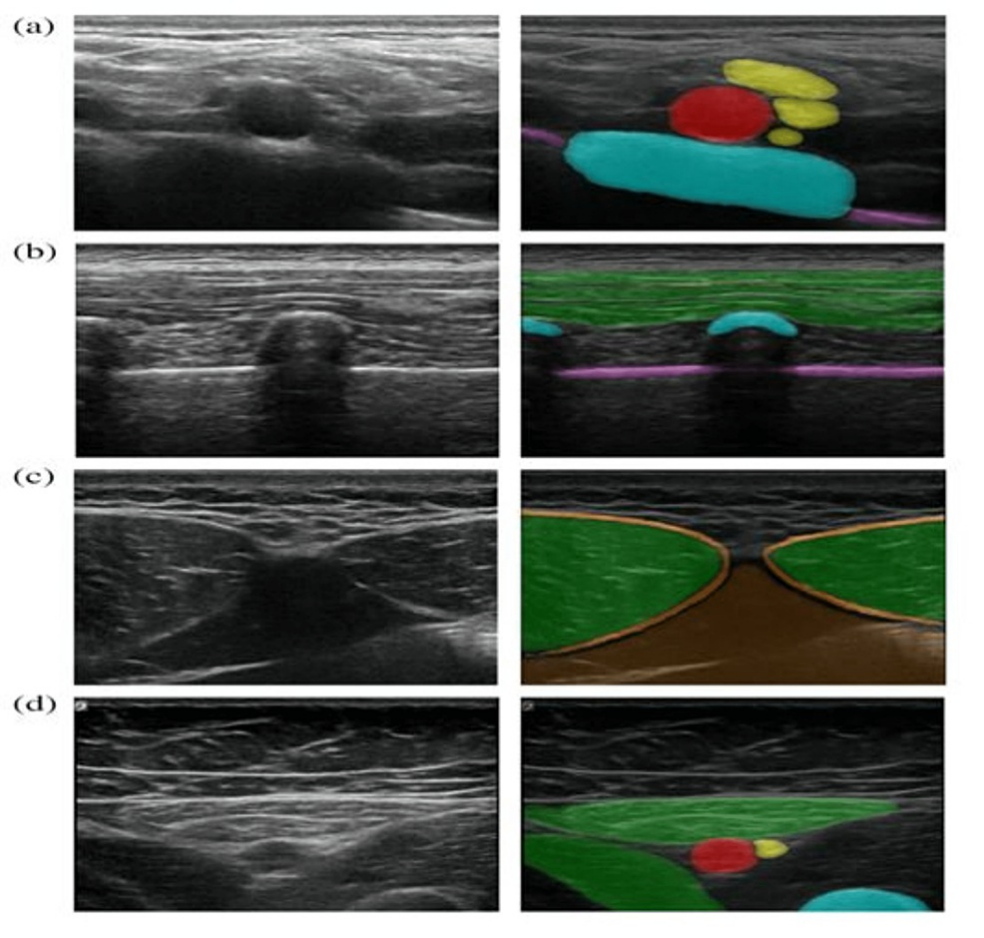
PNB regions and structures. PNB regions and structures color-overlayed by ScanNav™ [6]. (a) Supraclavicular-level brachial plexus: subclavian artery (red), brachial plexus nerves (yellow), first rib (blue), and pleura (purple). (b) Erector spinae plane (thoracic region): trapezius/rhomboid/erector spinae (group) muscles (green), vertebral transverse process/rib (blue), and pleura (purple). (c) Rectus sheath: rectus abdominis muscle (green), rectus sheath (orange), and peritoneal contents (brown). (d) Adductor canal: femoral artery (red), saphenous nerve (yellow), sartorius/adductor longus (green), and femur (blue). PNB: peripheral nerve block

Segmentation with color overlays describes the delineation of closely approximated structures by using color differentiation (Figure 3). Normal ultrasound images do not have color but different contrast intensities depending on the tissues scanned, which range from anechoic (does not reflect or transmit ultrasound waves) to hyperechoic (easily transmits or reflects ultrasound waves) [7]. To enhance contrast using color, an AI system called ScanNav™ Anatomy Peripheral Nerve Block (also known as ScanNav™ Anatomy PNB and formerly known as Anatomy Guide; Intelligent Ultrasound Ltd. {IUL}, Cardiff, United Kingdom) was developed (Figure 3). This system used deep convolutional neural networks (CNNs) to perform semantic segmentation of the ultrasound images provided. A study to determine the utility of ScanNav™ was undertaken where 40 ultrasound images each were acquired from known PNB views (Table 1) in patient volunteers when using ultrasound-guided regional anesthesia (UGRA) (Figure 4) [6]. These were color-segmented and then assessed by three independent experts in four key domains: 1) Did the video contain clinically relevant images of the block area? 2) What is the overall performance of highlighting? 3) Did highlighting help identify the concerned structure? 4) Would highlighting help confirm the correct ultrasound view to a practitioner with less experience? The system's overall performance was 7.87 out of a maximum score of 10. Also, highlighting was considered helpful in the identification of anatomical structures in 99.7% of the assessments, and it was considered helpful in confirming the correct view in 99.3% of assessments.

**Table 1:** PNB regions of interest and structures. PNB regions of interest and structures that were color-overlayed by ScanNav™^ ^[6]. PNB: peripheral nerve block

Serial number	PNB region	Structures in each block region that were highlighted
1	Interscalene-supraclavicular-level brachial plexus block	Subclavian artery, brachial plexus nerves (roots: trunks/divisions), sternocleidomastoid/anterior scalene muscles, first rib, and pleura
2	Axillary-level brachial plexus	Axillary artery, radial/median/ulnar/musculocutaneous nerve, fascia over the conjoint tendon of latissimus dorsi/teres major, and humerus
3	Erector spinae plane	Trapezius/rhomboid/erector spinae (group) muscles, ribs/thoracic vertebral transverse processes, and pleura
4	Rectus sheath	Rectus abdominis/transversus abdominis muscles, rectus sheath, and peritoneum/peritoneal contents
5	Suprainguinal fascia iliaca	Deep circumflex iliac artery, iliacus muscle, fascia iliaca, and ilium (anterior superior iliac spine)
6	Adductor canal	Femoral artery, saphenous nerve, sartorius muscle, adductor longus, and femur
7	Popliteal-level sciatic nerve	Popliteal artery and sciatic/tibial/common peroneal (fibular) nerves

**Figure 5:**
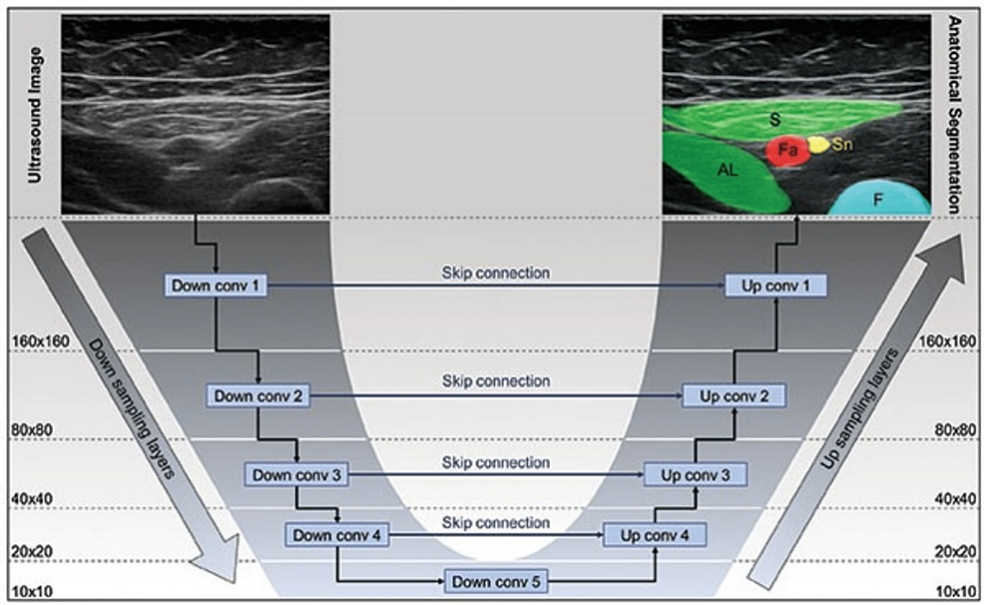
Deep learning methodology of ScanNav™. ScanNav™^ ^employs advanced deep learning using U-Net architecture (this is achieved through the use of convolutional neural networks {CNNs; ConvNets}) to identify anatomical structures in real-time ultrasound. The process involves down sampling to extract features and up sampling to refine details, with "skip connections" aiding data flow. Supervised learning involves pairs of unmodified and segmented images for each anatomical area. Over 800000 images were used [9].

The technical details of how ScanNav™ accomplished the image processing and color relaying are detailed in Figure 5 [6,8,9]. ScanNav™ underwent a further external validation study in 2022 when 720 ultrasound assessments were done in 40 patient subjects, which were then color-overlayed [5]. Three UGRA experts reviewed the color overlays for anatomical accuracy and the potential of reducing the risk of injury or adverse effects to the patients if PNB had been performed. A total of 1680 key anatomical structures were assessed each by three expert reviewers. The reviewers reached a consensus opinion in 1624 of the 1680 structures (96.7%); however, no consensus was reached in 56 structures (3.3%). The authors of the study found that ScanNav™ identified significant anatomical structures in 93.5% of cases. Color overlays also reduced the risk of unwanted needle trauma to nerves, arteries, pleura, and peritoneum in 62.9%-86.4% of cases and also increase the risk in 0.0%-1.7% of cases (Table 2). The risk of block failure was reported to be reduced in 81.3% of scans and increased in 1.8%.

**Table 2:** PNB regions with surrounding structures at risk of injury. PNB regions with surrounding structures at risk of injury that were color-overlayed by ScanNav™ [5]. PNB: peripheral nerve block

Serial number	PNB region	Nerve	Artery	Serosal plane	Bone	Fascia
1	Interscalene-level brachial plexus block	C5 and C6 nerve roots				
2	Upper trunk block	Upper trunk of the brachial plexus				
3	Supraclavicular-level brachial plexus block	Trunks/divisions of the brachial plexus	Subclavian artery	Pleura		
4	Axillary-level brachial plexus block	Musculocutaneous, median, ulnar, and radial nerves	Axillary artery			
5	Erector spinae plane block			Pleura	Transverse process	
6	Rectus sheath block			Peritoneum		Rectus sheath
7	Suprainguinal fascia iliaca block		Deep circumflex iliac artery			Fascia iliaca
8	Adductor canal block	Saphenous nerve	Femoral artery			
9	Popliteal-level sciatic nerve block	Sciatic nerve	Popliteal artery			

With increasing confidence in the use of AI in anatomical guidance, the FDA recently approved ScanNav™ for use in the USA [10].

**Figure 6:**
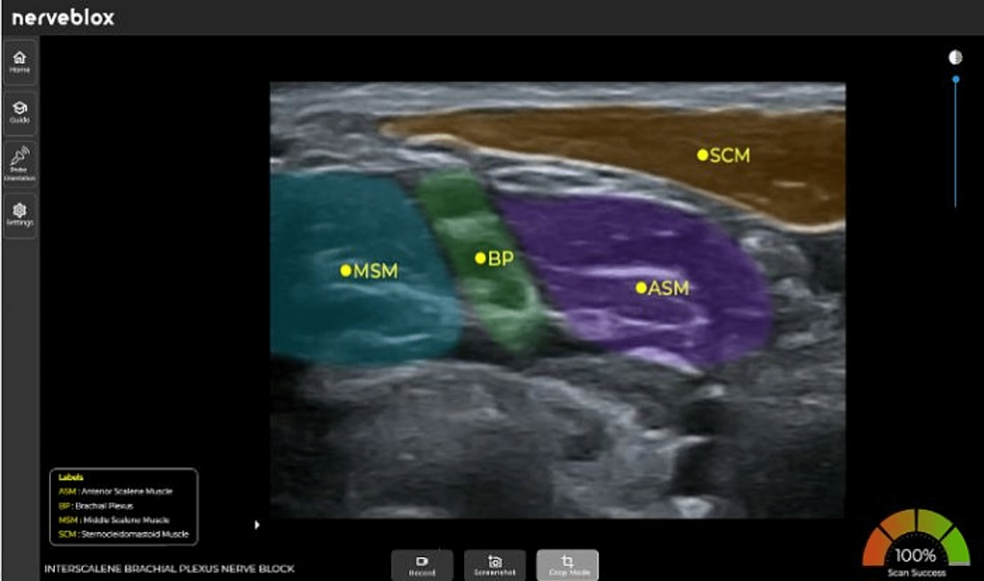
NerveBlox® AI technology showing the boundary prediction of interscalene block. NerveBlox® AI technology showing the boundary prediction of interscalene block with predefined anatomical landmarks in the color-labeled overlay. Permission granted by NerveBlox® (SmartAlpha, Ankara, Turkey), which is an artificial intelligence software as a medical device (SaMD) with CE marking. MSM, middle scalene muscle; ASM, anterior scalene muscle; BP, brachial plexus; SCM, sternocleidomastoid muscle; AI, artificial intelligence

NerveBlox® (Figure 6) is another AI software that can identify anatomical landmarks and provide color overlays. NerveBlox® presents a "scan success" rate expressed as a percentage. A 100% "scan success" rate indicates confidence in the labeling of anatomical landmarks when scanning a PNB region.

Its use was validated in a study in both male and female volunteer patients for interscalene, supraclavicular, infraclavicular, and transverse abdominal plane blocks. These scans were performed by three trainees on each healthy volunteer. The targeted regions were scanned, and the freeze function was used when "scan success" was 100%, and the corresponding raw US image with generated labels was saved. A total of 480 raw and labeled image pairs were obtained from 40 healthy volunteers. The images were assessed for the accuracy of labeling by a blinded external anesthesiologist and radiologist. As per the study, there was good consistency in labeling the landmarks, and accuracy was achieved in both male and female patient subjects (Table 3) [11].

**Table 3:** Summary of the studies utilizing AI in regional anesthesia. UGRA, ultrasound-guided regional anesthesia; IQR, interquartile range

Author/year	Aim	Sample	Methods	Outcome
Bowness et al./2021	To evaluate the performance of an assistive artificial intelligence (AI) system in aiding the identification of anatomical structures on ultrasound	One hundred forty-four anonymized ultrasound videos were recorded from patients undergoing treatment at four clinical centers	Three experts evaluated 40 ultrasound scans of seven body areas, comparing AI-enhanced and unmodified videos and assessing system performance and anatomical structure identification. Two hundred seventy-five evaluations were conducted; average highlighting scores ranged from 7.87 to 8.69 (out of 10)	AI highlighting was helpful in identifying specific anatomical structures in 1330/1334 cases (99.7%) and for confirming the correct ultrasound view in 273/275 scans (99.3%)
Bowness et al./2022	To examine AI's role (ScanNav™) in ultrasound image acquisition and interpretation by using a color overlay for anatomical structures	Two healthy volunteers for ultrasound scanning were recruited from a professional modeling agency. Half of the scans were performed on each subject	A total of 240 ultrasound scans were performed across nine nerve block regions, with 30 anesthesiologists, 15 non-experts, and 15 UGRA experts participating. Half of the scans used ScanNav™. After each scan, the participants completed a questionnaire, and both ultrasound and color overlay outputs were recorded. Real-time and remote experts assessed potential risks associated with ScanNav™, with differences in perception noted	Non-experts provided more positive feedback (p=0.001); 61.7% saw training potential. Experts were more focused on teaching (50%). Risk perception varied slightly between real-time and remote experts
Bowness et al./2023	To evaluate the accuracy of the artificial intelligence color overlay and its perceived influence on the risk of adverse events or block failure	Forty healthy adult subjects were recruited for scanning	Experts collected 720 videos (across nine regions), first without AI and then AI with color overlay. Three more experts reviewed each video to assess overlay accuracy, highlighting the impact on risk perception and block accuracy	AI models accurately identified the target structure in 93.5% of cases (1519/1624). False-negative rate was 3.0% (48/1624), and false-positive was 3.5% (57/1624), highlighting lowered needle trauma risk in 62.9%-86.4% of cases (from 302/480 to 345/400) and slightly increased in 0.0%-1.7% (from 0/160 to 8/480). The risk of block failure was reduced by 81.3% (585/720) and increased by 1.8% (13/720)
Scholzen et al./2023	To examine NerveBlox® AI software usability via self-reported questionnaires among trainees and faculty anesthesiologists in an academic medical center	Three healthy volunteers from the University of Wisconsin School of Medicine and Public Health's standardized patient group participated, receiving compensation. One patient was available throughout the day, while the other two shared their time equally. The participants included one female and two males	At the University of Wisconsin, 11 anesthesiologists and 25 residents simulated nerve blocks using NerveBlox® software on standardized patients. A survey gauged the software's utility and functionality	Both faculty and resident anesthesiologists rated NerveBlox® software's ability to identify anatomical structures at 8 (resident IQR: 8-9, n=25; faculty IQR: 6.5-9.5, n=11). They also highly valued the software's usefulness as a teaching aid, with residents rating it at 10 (IQR: 9-10, n=25) and faculty at 9 (IQR: 7.5-10, n=11). The "scan success" feature was also well-received, rated 8 and 9 by residents and faculty, respectively, for both performing and teaching regional anesthesia

Training and Education

In our experience, the difficulty of acquiring experience and skills in the safe performance of UGRA is due to four major factors; most trainees do not possess sufficient knowledge of clinically relevant anatomy as it pertains to ultrasound-guided nerve blocks [12]. Patient safety precludes multiple attempts to practice on a live patient, thereby establishing the need for high-fidelity simulator training [1,13], and inability to translate the 3D-orientated experience to traditional two-dimensional (2D) textbooks and videos [14], and interpreting traditional ultrasound images even in the presence of a supervisor can be daunting as the human eye is not trained to easily differentiate echogenic structures.

The use of AI has allowed for new methods of teaching and training in UGRA. Machine learning (ML) allows the consumption and processing of substantial amounts of raw data and the identification of recognizable patterns. This in turn allows AI to determine the chances of the success of a procedure by considering multiple variables such as patient position, injection site, needle positioning, and the evaluation of the dose and spread of local anesthetic to increase the chance of success [15]. The trainee doctor can receive feedback on their performance. With repeated performance, the trainee can improve.

The ability of ScanNav™ to introduce color overlays of important structures, as well as the ability to confirm PNB regions, increases operator confidence. In one study, ultrasound scans were performed with ScanNav™ involving 15 non-expert anesthesiologists and 15 experts in UGRA. Non-experts and experts were then asked to review their experience [16]. Non-experts completed a questionnaire on the utility of the device in relation to training, teaching, and clinical practice. Experts assessed the device for the potential for increased risk with use (such as needle trauma to the nerve causing nerve injury, to the pleura causing pneumothorax, and to the peritoneum causing peritoneum injury; local anesthetic systemic toxicity; or block failure). In addition, other parameters such as confidence in their own scanning, clarity in identifying anatomical structures, teaching scanning for the block, supervising scanning for the block, and confidence in non-expert's scanning ability were assessed. A total of 240 scans had been performed (120 with ScanNav™ and 120 without ScanNav™). Non-experts provided positive feedback more often and negative feedback less frequently than experts. The most frequent positive feedback given by non-experts was on the role of ScanNav™ in training. Experts gave positive feedback most frequently on its role in teaching. However, non-experts' most frequent negative feedback was that it may decrease their confidence in scanning and for experts was that it may increase the frequency of supervisor intervention. Experts also assessed that using ScanNav™ had a potential increase in the patient risk of 4.7% versus 3.1% without ScanNav™.

Similarly, in another study, 11 anesthesiologists and 25 anesthesiology trainees used the NerveBlox® software and found it to be helpful in performing anesthesia procedures and improving regional anesthesia education [17].

An interesting study on anatomical training using AI-assisted nerve identification based on a convolutional neural network was conducted at Beijing Jishuitan Hospital [18]. A total of 40 trainees who had theoretical training in performing sciatic nerve blocks were randomized to either a traditional teaching group or an AI teaching group. The trainees in the traditional teaching group were then asked to practice ultrasound operations on a mannequin under the guidance of an instructor. They were then allowed to practice US-guided puncture on a nerve block simulator. An AI-assisted identification system for ultrasound-guided nerve block was established based on the convolutional neural network by acquiring videos of US-guided sciatic nerve block. The videos were decomposed into multiple frames, which were then labeled by 15 experienced doctors. The AI algorithm studied the labeling using deep learning. A total of 1721 images were used to train and validate the AI-assisted identification system. The residents randomized to the AI teaching group were asked to practice marking the sciatic, tibial, and common peroneal nerve on an iPad Air (Apple, Inc., Cupertino, CA) using an Apple Pencil (Apple, Inc., Cupertino, CA) on the same images that were used to train the AI. These labels were then checked for intersection by AI-guided labeling. The extent of the intersection was scored, and after scoring above 80 points, the residents were allowed to practice US-guided puncture training on a nerve block simulator. Both groups were then allowed to perform popliteal nerve blocks in patients, and their performance was assessed. The residents that trained in the traditional teaching group were allowed to clarify any anatomical query from the teachers of the research group. The residents from the AI teaching group were allowed to record an ultrasound (US) video and import it into the system and use AI identification for help if they had questions. It was found that residents in the AI teaching group had significantly lower complication rates of paresthesia during puncture.

The use of augmented reality (AR) is another scenario where AI can be involved in teaching and training by creating virtual simulations for the trainee to practice and receive feedback. Such systems can also be made high fidelity with haptic feedback [19]. Kim and Tsui have suggested that using simulation would shorten the learning curve of trainees and improve block success [13]. Additionally, it would improve communication and situational awareness and reduce stress. AR can be effective in teaching UGRA anatomy as an online virtual simulator and presenting 3D rendering of anatomy in virtual models. Simulation-based training also improves hand-eye coordination and improves dexterity. With the use of haptic feedback, the feel of the needle and tissue interaction can also be simulated.

Eye-tracking technology has been studied as a means of identifying performance attributes in experienced versus beginner proceduralists. It was found that compared to beginners who focus more on visually salient points (areas that stand out regardless of importance), experts focus more on cognitively salient points of foci (areas relevant to the performance of a task) [20]. In one published study, the authors opined that experts tend to show fewer fixations and fixate for a longer period in areas of interest compared to beginners who tend to shift their gaze more often and to more locations. Experts also fixate on a target for a longer period before taking action, which has been termed the "quiet eye period." In their study, they observed that experts readily aligned the needle and ultrasound plane while solely observing the ultrasound screen, whereas beginners fixate more on the hands and ultrasound transducer to achieve needle guidance. Eye tracking, therefore, offers a potential avenue to improve ultrasound education. AI by means of its computing power and rapid feedback can guide trainee anesthetists to utilize the "quiet eye period" and to deliberate more on techniques and maneuvers with a better chance of procedure success. The efficiency and economy of movements can be emphasized upon backed by real-time data.

Like eye tracking, hand motion analysis was also studied as a means of quantifying a difference in skill level between beginners and expert levels. A study was conducted on hand motion analysis using the Imperial College Surgical Assessment Device (ICSAD) in performing regional anesthesia. ICSAD was used to measure three dexterity parameters during the scanning and needling phases of each block: time taken, number of movements, and path length traveled by each hand. The researchers found that experts performed significantly better than residents in both the scanning and needling phases. Regional anesthesia fellows, who were also included in the study, showed improvement in their ICSAD parameters, indicating a transition to the expert level. ICSAD data can also be used to improve UGRA training [21].

It is potentially possible for all these technologies to come together to create a near-total immersive environment for the trainee to deliberately practice until they reach an adequate level of expertise.

Improved Patient Safety and Outcomes

AI in patient safety is an evolving topic. In regional anesthesia, the use of color overlays to label vital neighboring structures in a PNB region has been shown to reduce the risk of unwanted injury by the needle and reduce the risk of block failure [5]. The anatomical training of trainees who performed popliteal nerve block, using an AI labeling system based on the deep convolutional neural network, has been shown to reduce the incidence of paresthesia in patients [18].

Other potential applications in patient safety are shown in Table 4.

**Table 4:** The use of AI in patient safety. AI, artificial intelligence; US, ultrasound

Potential use of AI in patient safety while performing regional anesthesia
The analysis of electronic health records and issuing alerts: Local anesthesia allergy alert, previous history of local anesthesia systemic toxicity, and alert impaired coagulation
The analysis of real-time US image: Improved needle tip localization using color overlays to highlight significant surrounding structures such as the arteries, veins, nerves, pleura, and peritoneum. The correct identification of the peripheral nerve region corresponding to the block to be performed. The improved visualization of the spread of local anesthetic. Check for other pathologies that may impede nerve blocks such as fibrosis, inflammation, and fracture
Patient monitoring: Real-time ECG monitoring and arrhythmia detection during and after a peripheral nerve block. Checking for injection pressure and issuing an alert if pressure is high. Checking for blood aspiration and issuing an alert if blood is aspirated in a needle
Population health: The analysis of patient records and examination for population health outcomes (e.g., the use of femoral nerve block in geriatric population for hip replacement in a state and outcomes)

Robotic technology in regional anesthesia

The utilization of mechanical robots in anesthesia is still in its initial stages and has been mainly explored for tracheal intubation and regional anesthesia. An exemplary application is the Robotic Endoscope-Automated Laryngeal Imaging for Tracheal Intubation (REALITI), which enables real-time image recognition and automated orientation for intubation [22]. Initial tests on mannequins indicated that untrained individuals performed better with REALITI's automated mode than with manual control.

In another example, researchers have employed the DaVinci system to conduct experiments involving nerve blocks and the insertion of perineural catheters, using ultrasound guidance on nonhuman models [23].

Another system, the Magellan, is tailored for semiautomated peripheral nerve blocks through ultrasound guidance in regional anesthesiology. The robotic setup involves an arm bearing a nerve block needle at its tip, operable via a joystick and a software control system [24]. Moreover, a recent study utilized the Magellan system to explore learning curves [25]. With five anesthetists performing on a nerve phantom model, learning enhanced over 10 needle insertions compared to manual insertion. Limited by sample size and repetitions, this study highlighted the need for comprehensive training when adopting novel technology. Similar trends emerged in testing a regional block needle tip-tracking system, emphasizing the requirement for meticulous training during technology adoption [26].

However, preventing excessive reliance on robotic assistance during training can be a challenge. Although it may decrease variability among trainees, it might compromise overall competence. Such overreliance could pose risks during emergencies or equipment failure. Thus, designing robotic interventions as feedback systems to complement, not replace, the learning process is crucial. The technology's early learning curve reflects its novelty, warranting a balanced approach to its integration into anesthesia practice.

Upcoming clinical systems are poised to do more than just alert an anesthetist of issues; they could also propose or administer treatments. Cognitive robots, often referred to as clinical decision support (CDS) systems, can be categorized into two main types. The first is rule-based expert systems, which depend on algorithms crafted by specialists in the field. The second category comprises machine learning systems, which educate themselves by recognizing recurrent patterns in the data acquired throughout patient care procedures [27].

An illustrative instance is the SAFer Injection for Regional Anesthesia (SAFIRA), a recent medical device [28]. It eliminates the need for an assistant during nerve blocks while maintaining the ability to aspirate and halt injection if pressure surpasses 15 psi.

Robotic systems are likely to complement anesthesia practice, as they can handle multifaceted tasks such as understanding intricate medical backgrounds, monitoring vital signs, and making pivotal judgments during unusual circumstances. Soon, these systems might operate on autopilot until manual intervention is necessary, although ultimate clinical decisions will remain human-driven.

Exploring the role of AI in pain management

Pain management stands as a fundamental aspect of healthcare, aiming to alleviate suffering and enhance patients' quality of life. The emergence of artificial intelligence (AI) has brought new possibilities to the field, offering innovative tools and strategies for optimizing pain assessment, treatment, and overall patient outcomes.

AI technologies have shown promise in revolutionizing pain assessment and diagnosis. Using AI/ML methods to identify pain via facial expressions offers numerous benefits. It provides precise pain intensity measurements, aiding in accurate diagnosis and treatment. Moreover, it is invaluable for detecting pain in scenarios where assessment is challenging, such as nonverbal or critically ill patients and the perioperative phase [29-32]. For example, Fontaine et al. [31] evaluated postoperative pain using AI-based facial expression recognition. A ResNet-18 neural network was trained to classify 2810 facial expressions from 1189 patients, linked to self-reported pain intensity (0-10 scale). AI accuracy, sensitivity, and specificity to diagnose pain levels of ≥4/10 and ≥7/10 were evaluated. AI's performance was compared with 33 nurses' assessments. In external testing (120 images), AI predicted exact pain intensity in 53% of cases (error of ±2.4). Sensitivity for pain of ≥4/10 and ≥7/10 was 89.7% and 77.5%. Nurses' accuracy was 14.9%, with sensitivities of 44.9% and 17.0%. In conclusion, while AI facial expression analysis requires refining, it holds the potential to assist physicians in pain assessment, especially for noncommunicative individuals. This deeper understanding allows for more accurate and personalized pain management plans.

Through the integration of AI/ML tools, medical professionals can analyze and translate patients' facial expressions linked to pain. This empowers them to personalize treatments and medication dosages to suit individual requirements. Furthermore, having an unbiased and continuous way to monitor postoperative pain intensity could offer substantial benefits, potentially allowing accurate and cost-effective pain evaluation. Notably, certain research outcomes indicate that AI/ML surpasses human observers in distinguishing authentic pain from feigned expressions [33,34]. Thus, AI aids in precise pain assessment, empowering clinicians to make more informed decisions regarding the diagnosis and treatment of pain.

AI-incorporated applications exhibited positive impacts on pain management, such as reducing pain intensity, reducing the frequency of other interventions, and aiding therapeutic exercises [35-37]. However, the studies have several limitations. Some studies assessed general pain levels instead of specific site pain. Also, research only covered immediate post-intervention effects, leaving long-term effects uncertain. Investigating if pain reduction affects other functions or physiology is necessary. Application adherence was not evaluated. Additionally, comparing these interventions to routine pain care is vital to establish the benefits in adopting an innovative methodology to optimize pain assessment and management.

Ethics of AI in regional anesthesia and pain management

Despite AI's widespread utility and growing evidence of benefits in patient care, concerns have been raised about safety and accountability. AI systems are semiautonomous to fully autonomous. Their input, while validated by many studies, are also prone to error.

A cross-sectional online questionnaire-based survey was conducted among Turkish anesthesiologists to examine their opinions of the ethical implications of the use of AI in regional anesthesia [38]. Some of the questions asked included privacy concerns and who would be responsible for errors committed while using AI. Additionally, participants were asked to weigh beneficence and maleficence to patients. The majority expressed that AI would benefit patients and medical education. They also expressed that privacy is not a concern if data is captured anonymously. However, a majority also expressed concern about responsibility if errors were to occur.

Other colleagues have expressed that AI systems are only as good as the quality of data fed into them, highlighting concerns for inaccuracies that may happen if the input data also has errors [39]. Additionally, governance and regulations on how to manage data, patient privacy, and medicolegal concerns have been discussed. It is a unanimous opinion that AI has strong benefits but needs a strong regulatory body to assess how it should move forward and how might AI be incorporated into the normal workflow. Concerns about patient privacy and responsibility must be clearly addressed. Transparent reporting, continuous appraisal, and the upgradation of systems are required. As the use of AI becomes more common, more real-world data will become available, which will guide us on how to move forward.

The Alan Turing Institute, which deals with data science and artificial intelligence, provides guidance on AI ethics and safety. It advocates principles of fairness, accountability, sustainability, and transparency [40,41]. The key themes for safe and ethical AI as per the Alan Turing Institute include the following: advancing appropriate AI transparency and applicability, improving the fairness of algorithmic systems, including ways to measure and mitigate bias, developing robust systems that adapt well to new environments secure from attack, respecting privacy, developing systems that work effectively together with humans, maintaining appropriate human control, and preventing undue influence.

It is the view of the authors, however, that despite AI assistance, the responsibility for the safe performance of the procedure should rest solely on the anesthetist alone and should by no means be delegated to an autonomous replacement.

Challenges in applying AI in regional anesthesia

Ultrasound-guided procedures face challenges such as altered illumination, occlusion, artifacts, and noise, leading to tracking failure and false alarms. Sudden motion changes and object shape alteration further complicate accurate tracking. These factors can lead to tracking failure and false positives, potentially misleading medical practitioners and compromising procedure efficacy.

Even with AI assistance, there is a chance of AI misidentifying anatomy. A firm grasp of sonographic anatomy remains crucial. Hence, in ultrasound-guided regional anesthesia (UGRA) training, experienced educators play a vital role, and "AI-assisted devices" should not replace them. Trainees should still focus on mastering conventional sonographic scanning techniques, probe manipulation, and image acquisition. Furthermore, the effectiveness of AI-supported UGRA could be assessed through various measures, including precision, reliability, time efficiency, noise resilience, cost-effectiveness, and, frequently, human-based qualitative assessment of visual outcomes. Therefore, future trials should answer these challenges to quantify AI's role in the field of regional anesthesia.

This focused review aimed to explore the range of artificial intelligence research in regional anesthesia and pain management. Unlike a systematic review that addresses AI's clinical efficacy, this study highlights AI's potential implications, clinicians' role in its adoption, and its limitations. Although some relevant papers might not have been covered by our search criteria, this review's breadth underscores the areas AI could impact for clinicians and its limitations.

## Conclusions

In conclusion, AI aids in identifying structures on ultrasound images. Given the frequent use of ultrasound visualization in regional anesthesia, AI solutions could prove valuable in identifying anatomical landmarks. This has the potential to minimize complications, including inadvertent structural injury and systemic toxicity from local anesthetics. AI-guided approaches enhance sonographic image interpretation, needle advancement visualization, and local anesthetic injection precision. They might also elevate the training process for ultrasound-guided regional anesthesia (UGRA). Interestingly, these discoveries suggest that AI, particularly through machine learning, has the potential to simplify pain evaluation and self-care. Yet, there remains ample scope to advance in predicting pain and crafting AI-driven systems to aid pain treatment. Thus, future studies warrant robust randomized controlled trials with large sample sizes and systematic reviews and meta-analyses.

## References

[1] Smith AF, Pope C, Goodwin D, Mort M (2006). What defines expertise in regional anaesthesia? An observational analysis of practice. Br J Anaesth.

[2] Sites BD, Spence BC, Gallagher JD, Wiley CW, Bertrand ML, Blike GT (2007). Characterizing novice behavior associated with learning ultrasound-guided peripheral regional anesthesia. Reg Anesth Pain Med.

[3] Tenajas R, Miraut D, Illana CI, Alonso-Gonzalez R, Arias-Valcayo F, Herraiz JL (2023). Recent advances in artificial intelligence-assisted ultrasound scanning. Appl Sci.

[4] Henderson M, Dolan J (2016). Challenges, solutions, and advances in ultrasound-guided regional anaesthesia. BJA Educ.

[5] Bowness JS, Burckett-St Laurent D, Hernandez N (2023). Assistive artificial intelligence for ultrasound image interpretation in regional anaesthesia: an external validation study. Br J Anaesth.

[6] Bowness J, Varsou O, Turbitt L, Burkett-St Laurent D (2021). Identifying anatomical structures on ultrasound: assistive artificial intelligence in ultrasound-guided regional anesthesia. Clin Anat.

[7] Brull R, Macfarlane AJ, Tse CC (2010). Practical knobology for ultrasound-guided regional anesthesia. Reg Anesth Pain Med.

[8] Μoka Ε, Bowness J (2021). Artificial intelligence and robotics in regional anaesthesia: do they have a role?. Signa Vitae.

[9] Bowness J, Macfarlane AJ, Noble JA, Higham HA, Burckett-St Laurent D (2021). Anaesthesia, nerve blocks and artificial intelligence. Anaesth News.

[10] Larkin HD (2022). FDA approves artificial intelligence device for guiding regional anesthesia. JAMA.

[11] Gungor I, Gunaydin B, Oktar SO, M Buyukgebiz B, Bagcaz S, Ozdemir MG, Inan G (2021). A real-time anatomy ıdentification via tool based on artificial ıntelligence for ultrasound-guided peripheral nerve block procedures: an accuracy study. J Anesth.

[12] Mongodi S, Bonomi F, Vaschetto R (2022). Point-of-care ultrasound training for residents in anaesthesia and critical care: results of a national survey comparing residents and training program directors' perspectives. BMC Med Educ.

[13] Kim TE, Tsui BC (2019). Simulation-based ultrasound-guided regional anesthesia curriculum for anesthesiology residents. Korean J Anesthesiol.

[14] Worm BS, Krag M, Jensen K (2014). Ultrasound-guided nerve blocks--is documentation and education feasible using only text and pictures?. PLoS One.

[15] Shorten G, Srinivasan KK, Reinertsen I (2018). Machine learning and evidence-based training in technical skills. Br J Anaesth.

[16] Bowness JS, El-Boghdadly K, Woodworth G, Noble JA, Higham H, Burckett-St Laurent D (2022). Exploring the utility of assistive artificial intelligence for ultrasound scanning in regional anesthesia. Reg Anesth Pain Med.

[17] Scholzen EA, Schroeder KM (2023). Use of artificial intelligence software helpful for regional anesthesia education in self-reported questionnaire in academic medical center setting. Res Sq.

[18] Cai N, Wang G, Xu L (2022). Examining the impact perceptual learning artificial-intelligence-based on the incidence of paresthesia when performing the ultrasound-guided popliteal sciatic block: simulation-based randomized study. BMC Anesthesiol.

[19] Shevlin SP, Turbitt L, Burckett-St Laurent D, Macfarlane AJ, West S, Bowness JS (2023). Augmented reality in ultrasound-guided regional anaesthesia: an exploratory study on models with potential implications for training. Cureus.

[20] Harrison TK, Kim TE, Kou A, Shum C, Mariano ER, Howard SK (2016). Feasibility of eye-tracking technology to quantify expertise in ultrasound-guided regional anesthesia. J Anesth.

[21] Chin KJ, Tse C, Chan V, Tan JS, Lupu CM, Hayter M (2011). Hand motion analysis using the imperial college surgical assessment device: validation of a novel and objective performance measure in ultrasound-guided peripheral nerve blockade. Reg Anesth Pain Med.

[22] Biro P, Hofmann P, Gage D (2020). Automated tracheal intubation in an airway manikin using a robotic endoscope: a proof of concept study. Anaesthesia.

[23] Tighe PJ, Badiyan SJ, Luria I, Boezaart AP, Parekattil S (2010). Technical communication: robot-assisted regional anesthesia: a simulated demonstration. Anesth Analg.

[24] Hemmerling TM, Taddei R, Wehbe M, Cyr S, Zaouter C, Morse J (2013). Technical communication: first robotic ultrasound-guided nerve blocks in humans using the Magellan system. Anesth Analg.

[25] Morse J, Terrasini N, Wehbe M, Philippona C, Zaouter C, Cyr S, Hemmerling TM (2014). Comparison of success rates, learning curves, and inter-subject performance variability of robot-assisted and manual ultrasound-guided nerve block needle guidance in simulation. Br J Anaesth.

[26] McKendrick M, Sadler A, Taylor A (2021). The effect of an ultrasound-activated needle tip tracker needle on the performance of sciatic nerve block on a soft embalmed Thiel cadaver. Anaesthesia.

[27] Nathan N (2020). Rise of the machines: autonomous robotic systems in anesthetic practice. Anesth Analg.

[28] Bodhey A, Nair A, Seelam S (2023). SAFIRA pump: a novel device for fixed injection pressure and to control local anesthetic injection during peripheral nerve block. J Anaesthesiol Clin Pharmacol.

[29] Rahu MA, Grap MJ, Cohn JF, Munro CL, Lyon DE, Sessler CN (2013). Facial expression as an indicator of pain in critically ill intubated adults during endotracheal suctioning. Am J Crit Care.

[30] Sikka K, Ahmed AA, Diaz D, Goodwin MS, Craig KD, Bartlett MS, Huang JS (2015). Automated assessment of children’s postoperative pain using computer vision. Pediatrics.

[31] Fontaine D, Vielzeuf V, Genestier P (2022). Artificial intelligence to evaluate postoperative pain based on facial expression recognition. Eur J Pain.

[32] Wu CL, Liu SF, Yu TL (2022). Deep learning-based pain classifier based on the facial expression in critically ill patients. Front Med (Lausanne).

[33] Littlewort GC, Bartlett MS, Lee K (2009). Automatic coding of facial expressions displayed during posed and genuine pain. Image Vis Comput.

[34] Bartlett MS, Littlewort GC, Frank MG, Lee K (2014). Automatic decoding of facial movements reveals deceptive pain expressions. Curr Biol.

[35] Rabbi M, Aung MS, Gay G, Reid MC, Choudhury T (2018). Feasibility and acceptability of mobile phone-based auto-personalized physical activity recommendations for chronic pain self-management: pilot study on adults. J Med Internet Res.

[36] Sandal LF, Bach K, Øverås CK (2021). Effectiveness of app-delivered, tailored self-management support for adults with lower back pain-related disability: a selfBACK randomized clinical trial. JAMA Intern Med.

[37] Piette JD, Newman S, Krein SL (2022). Patient-centered pain care using artificial intelligence and mobile health tools: a randomized comparative effectiveness trial. JAMA Intern Med.

[38] Koçer Tulgar Y, Tulgar S, Güven Köse S, Köse HC, Çevik Nasırlıer G, Doğan M, Thomas DT (2023). Anesthesiologists’ perspective on the use of artificial intelligence in ultrasound-guided regional anaesthesia in terms of medical ethics and medical education: a survey study. Eurasian J Med.

[39] Cascella M, Tracey MC, Petrucci E, Bignami EG (2023). Exploring artificial intelligence in anesthesia: a primer on ethics, and clinical applications. Surgeries.

[40] McKendrick M, Yang S, McLeod GA (2021). The use of artificial intelligence and robotics in regional anaesthesia. Anaesthesia.

[41] (2023). The Alan Turing Institute: artificial intelligence (safe and ethical). https://www.turing.ac.uk/research/research-programmes/artificial-intelligence-ai/safe-and-ethical.

